# 

**Published:** 1872-09

**Authors:** 


☞ *The Editors are not responsible for the views of Contributors.*

